# Automated analysis of finger blood pressure recordings provides insight in determinants of baroreflex sensitivity and heart rate variability—the HELIUS study

**DOI:** 10.1007/s11517-023-02768-4

**Published:** 2023-01-23

**Authors:** D. Collard, B. E. Westerhof, J. M. Karemaker, W. J. Stok, P. G. Postema, C. T. P. Krediet, L. Vogt, B. J. H. van den Born

**Affiliations:** 1 Department of Internal Medicine, Section Vascular Medicine, Amsterdam Cardiovascular Sciences, Amsterdam UMC, University of Amsterdam, PO box 22660, 1100 DD Amsterdam, The Netherlands; 2 Department of Pulmonary Medicine, Amsterdam Cardiovascular Sciences, Amsterdam UMC, Vrije Universiteit Amsterdam, Amsterdam, The Netherlands; 3 Department of Medical Biology, Section Systems Physiology, Amsterdam UMC, University of Amsterdam, Amsterdam, The Netherlands; 4 Department of Cardiology, Amsterdam Cardiovascular Sciences, Heart Center, Amsterdam UMC, University of Amsterdam, Amsterdam, The Netherlands; 5 Department of Internal Medicine, Section Nephrology, Amsterdam Cardiovascular Sciences, Amsterdam UMC, University of Amsterdam, Meibergdreef 9, Amsterdam, The Netherlands; 6 Department of Public and Occupational Health, Amsterdam Public Health Research Institute, Amsterdam UMC, University of Amsterdam, Amsterdam, The Netherlands

**Keywords:** HRV, BRS, Sympathovagal balance, Cohort study, Algorithms

## Abstract

Sympathovagal balance is important in the pathogenesis of hypertension and independently associated with mortality. We evaluated the value of automated analysis of cross-correlation baroreflex sensitivity (xBRS) and heart rate variability (HRV) and its relationship with clinical covariates in 13,326 participants from the multi-ethnic HELIUS study. Finger blood pressure (BP) was continuously recorded, from which xBRS, standard deviation of normal-to-normal intervals (SDNN), and squared root of mean squared successive difference between normal-to-normal intervals (RMSDD) were determined. A subset of 3356 recordings > 300 s was used to derive the minimally required duration by comparing shortened to complete recordings, defined as intraclass correlation (ICC) > 0.90. For xBRS and SDNN, 120 s and 180 s were required (ICC 0.93); for RMSDD, 60 s (ICC 0.94) was sufficient. We included 10,252 participants (median age 46 years, 54% women) with a recording > 180 s for the regression. xBRS, SDNN, and RMSDD decreased linearly up to 50 years of age. For xBRS, there was a signification interaction with sex, with for every 10 years a decrease of 4.3 ms/mmHg (95%CI 4.0–4.6) for men and 5.9 ms/mmHg (95%CI 5.6–6.1) for women. Using splines, we observed sex-dependent nonlinearities in the relation with BP, waist-to-hip-ratio, and body mass index. Future studies can help unravel the dynamics of these relations and assess their predictive value.

**Graphical Abstract:**

Panel 1 depicts automatic analysis and filtering of finger BP recordings, panel 2 depicts computation of xBRS from interpolated beat to beat data of systolic BP and interbeat interval, and (IBI) SDNN and RMSDD are computed directly from the filtered IBI dataset. Panel 3 depicts the results of large-scale analysis and relation of xBRS with age, sex, blood pressure and body mass index.

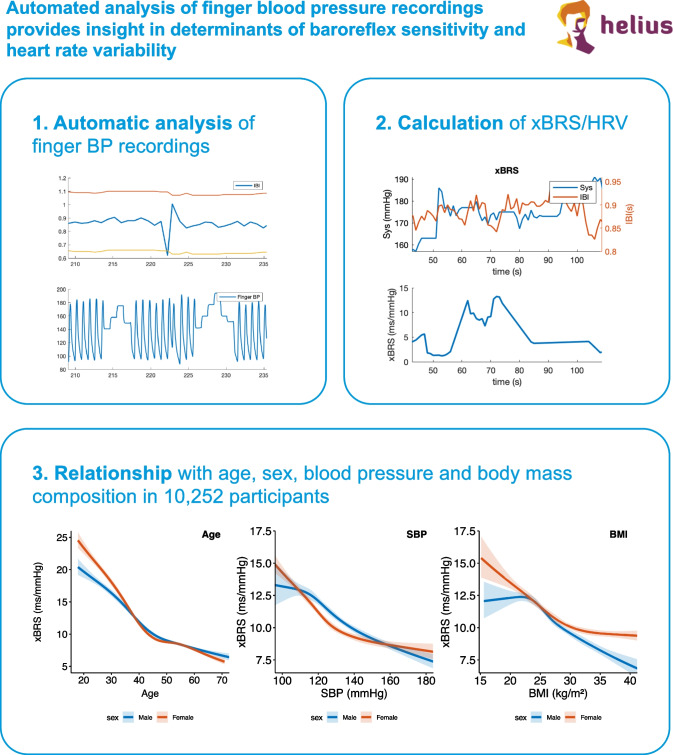

**Supplementary Information:**

The online version contains supplementary material available at 10.1007/s11517-023-02768-4.

## Introduction

Sympathetic and parasympathetic autonomic control, or sympathovagal balance, is important in the pathogenesis of hypertension and cardiovascular disease (CVD). Sympathovagal balance can be quantified by measurement of the cross-correlation baroreflex sensitivity (xBRS) and heart rate variability (HRV). Alterations in HRV and xBRS towards more sympathetic and less parasympathetic control are predictive of future cardiovascular events in subjects with and without established CVD [1]. The baroreflex is directly involved in the regulation of blood pressure, and changes can precede the development of hypertension and its complications [2, 3]. Additionally, increased sympathetic activity has been associated with other disease states leading to cardiovascular morbidity, including metabolic syndrome, obesity, and low-grade inflammation [4, 5].

Automatic analysis of these parameters using continuous non-invasive blood pressure recordings enables assessment of the sympathovagal balance in large-scale cohort studies and may provide additional insight in disease pathogenesis, treatment, and outcome prediction. The gold standard for the assessment of BRS is by measuring the heart rate response to pharmacologically induced blood pressure alterations. We have recently shown that it corresponds well to the cross-correlation BRS determined by cross-correlation of spontaneous variations in BP to changes in heart rate [6]. However, the performance of current algorithms to determine HRV and (x)BRS from continuous finger BP recordings at large scale remains to be determined. In this study, we developed a method to automatically process continuous BP tracings to allow for an integrated assessment of xBRS and short-term HRV and tested its accuracy in a large population-based study. Thereafter, we applied our automated analysis of xBRS and HRV to the complete dataset and assessed its relation with age, sex, blood pressure, and body composition.

## Methods

### Study design and measurements

For the present analysis, we used baseline data from the ongoing multi-ethnic HEalthy LIfe in an Urban Setting (HELIUS) prospective cohort study, which has been described in detail elsewhere [7]. In brief, 24,789 participants aged between 18 and 70 were included between 2011 and 2015. They were invited after random sampling stratified by ethnicity (Dutch, South-Asian Surinamese, African Surinamese, Ghanaian, Turkish, Moroccan origin) from the municipality register of the city of Amsterdam. All participants provided written informed consent and the study was approved by the medical research ethics committee of the Amsterdam UMC, location AMC, and followed the principles of the Declaration of Helsinki. The HELIUS data are owned by the Amsterdam UMC, location AMC, in Amsterdam, the Netherlands. Any researcher can request the data by submitting a proposal as outlined at http://www.heliusstudy.nl.

Because of logistical reasons, non-invasive continuous blood pressure recordings were performed in a subset including 13,326 participants. Five-minute recordings were taken in a supine position after at least 10 min of rest using the Nexfin device (Edwards Lifesciences, Irvine, CA). Study visits were conducted in the morning after an overnight fast and participants were asked to refrain from smoking prior to the visit. Office BP was determined by taking the average of two seated measurements performed using a validated semi-automatic oscillometric device (Microlife WatchBP Home; Microlife AG, Switzerland). Hypertension was defined according to current guidelines as either an elevated systolic (> 140 mmHg) or diastolic (> 90 mmHg) blood pressure or the self-reported use of blood pressure-lowering medication [8]. Diabetes was defined based on fasting plasma glucose levels (> 7.0 mmol/L) or the use of glucose-lowering medication. Anthropometric measurements, consisting of weight, height, waist circumference at the level midway between the lower rib margin and the iliac crest, and hip circumference at the widest point over the trochanter major were performed twice. Using the average, body mass index (BMI) and waist-hip ratio (WHR) were calculated. History of cardiovascular events was defined based on self-reported stroke, myocardial infarction, and coronary or peripheral revascularization. In all participants, an electrocardiogram (ECG) was performed using a MAC 1600 System (GE Healthcare) of 10 s to identify the basic heart rhythm. All ECGs were analyzed using the Modular ECG analysis system program, which was manually verified by a cardiologist as previously published [9]. For the present analysis, participants not in sinus rhythm were excluded.

### Analysis of sympathovagal balance

For each participant, raw beat-to-beat data consisting of SBP values and inter-beat intervals (IBI) derived from the finger arterial blood pressure measurement were exported from the Nexfin device. These data were further analyzed using a custom-written script in Matlab (R2019a; The MathWorks, Inc.); an overview of the algorithm is given in supplementary Fig. 1. First, to account for measurement artifacts and ectopic beats, the dataset was filtered using a local moving median filter. Subsequently, the complete IBI data set was filtered by replacing each point with local median of the surrounding 9 beats, and the average IBI of this filtered set was determined. Then, each beat which was not within local median ± 25% of the mean IBI of the filtered set was excluded from the original IBI set to derive a normal-to-normal interval set. If there were > 20% removed beats, the recording was excluded. Also, if no segment of at least 30 beats without automated internal calibration (Physiocal) was available, the individual record was excluded because of insufficient quality according to the manufacturer’s guidelines [10]. During these calibration intervals, heart rate data was still available; for xBRS, data was interpolated as described below. From the filtered IBI set, the standard deviation of normal-to-normal intervals (SDNN) and the square root of the mean squared successive differences between adjacent normal-to-normal intervals (RMSDD) were determined to quantify HRV, following current standards [11]. The xBRS was determined by using the cross-correlation method as previously described [12]. To calculate xBRS, the filtered IBI and SBP dataset were interpolated at 1 Hz using piecewise cubic hermite polynomials (PCHIP). Using a sliding window, for each consecutive sample in the interpolated set, a 10-s long interval of IBI was created. Each 10-s interval of IBI was cross-correlated with a 10-s interval of SBP measurements, with a time shift varying between 0 and 5 s, where SBP precedes IBI. The time delay with maximum correlation was chosen, and the xBRS for this segment was determined by dividing the standard deviation (SD) of the IBI by the SD of the SBP for that segment. Then, the xBRS of the complete recording was calculated as the geometric mean of all segments with significant positive correlation (*p* < 0.05). The Matlab script used for the calculation of the parameters is made available in the online data supplement.

### Statistical analysis

We first performed an analysis of the subset of the participants with optimal recordings, based on a minimal length of 300 s after the attainment of a stable signal defined as an interval of at least 30 beats without automatic internal calibration and a maximum of 20% excluded beats. For this subset, HRV and xBRS were calculated using progressive shortened recordings starting from both the start and the end of the original recording. Time periods of 30 s, 60 s, 120 s, 180 s, 240 s, and 300 s were chosen to assess the minimally required duration by comparing the measurements with the complete recording using Bland–Altman analysis and ICC coefficients [13]. We considered an ICC > 0.90 to be indicative of an acceptable correlation [14, 15]. Recordings with a length of 180 s shortened from the beginning and from the back were compared using a paired *t*-test to assess systematic differences resulting from prolonged resting measurements. In addition, we analyzed a random subset of 100 records manually, where we selected an optimal segment between 180 and 300 s and visually inspected the recording for noise and other outliers. These were compared to the complete, automatically analyzed recording to verify the automatic analysis. Based on these findings, we used all the subset of recordings of sufficient length and quality for the second part of the analysis. Baseline characteristics of the subset with optimal recordings, recordings of sufficient quality, and recording of insufficient quality were subsequently compared to explore differences between groups (Table 1). In the complete set with recordings of sufficient quality, we then performed linear regression analyses using restricted cubic splines to examine associations of xBRS and HRV with age, SBP, BMI, and WHR. Given the earlier observed sex differences in autonomic function, we tested whether the relation was different between men and women by adding an interaction term for sex to each model [16]. For SBP, WHR, and BMI, we included a linear term for age in the model and depicted the results with respect to a mean age of 45 years. The outcome variables were winsorized by replacing outliers below and above to the 1st and 99th percentile with the 1st and 99th percentile, and the values of the xBRS were log-transformed prior to analysis. All statistical analyses were performed using R version 3.6.1. A *p*-value < 0.05 was considered to indicate statistical significance.

**Table 1:** Baseline characteristics for complete cohort; the subset of optimal quality used for derivation of the quality criteria and the subset of sufficient quality which was used for the regression analysis. *BP* blood pressure, *SBP* systolic blood pressure, *HR* heart rate, *BMI* body mass index, *WHR* waist hip ratio

	Overall	Optimal quality	Sufficient quality	Insufficient quality
n	13,326	3356	10,252	3074
Age, years (median [IQR])	46.0 [34.0, 54.0]	45.0 [33.0, 54.0]	46.0 [33.0, 54.0]	47.0 [35.0, 56.0]
Women, *n* (%)	7654 (57.4)	1850 (55.1)	5555 (54.2)	2099 (68.3)
Ethnicity, *n* (%)				
Dutch	2758 (20.7)	821 (24.5)	2042 (19.9)	716 (23.3)
South-Asian Surinamese	1838 (13.8)	256 (7.6)	1340 (13.1)	498 (16.2)
African Surinamese	2833 (21.3)	790 (23.5)	2076 (20.2)	757 (24.6)
Ghanaian	1761 (13.2)	348 (10.4)	1251 (12.2)	510 (16.6)
Turkish	1992 (14.9)	497 (14.8)	1728 (16.9)	264 (8.6)
Moroccan	2144 (16.1)	644 (19.2)	1815 (17.7)	329 (10.7)
Hypertension, *n* (%)	4483 (33.7)	1033 (30.9)	3455 (33.8)	1028 (33.5)
Diabetes, *n* (%)	1235 (9.3)	298 (8.9)	1008 (9.9)	227 (7.5)
Smoking, *n* (%)	3180 (24.0)	825 (24.7)	2505 (24.5)	675 (22.1)
History of cardiovascular event, *n* (%)	1950 (14.7)	445 (13.3)	1509 (14.8)	441 (14.4)
BP-lowering drugs, *n* (%)	2283 (17.1)	527 (15.7)	1701 (16.6)	582 (18.9)
Beta-blockers, *n* (%)	832 (6.2)	183 (5.5)	601 (5.9)	231 (7.5)
SBP, mmHg (mean (SD))	127.8 (17.7)	126.4 (17.2)	128.0 (17.6)	126.9 (18.3)
HR, beats per minute (mean (SD))	68.8 (10.2)	68.7 (10.0)	69.4 (10.2)	66.6 (9.8)
BMI, kg/m^2^ (mean (SD))	27.1 (5.2)	27.2 (5.2)	27.6 (5.3)	25.7 (5.0)
WHR (mean (SD))	0.90 (0.09)	0.90 (0.09)	0.91 (0.09)	0.89 (0.08)
Sinus rhythm on ECG, *n* (%)	12,909 (98.7)	3356 (100.0)	10,252 (100.0)	2657 (94.0)
Length of recording, s (median [IQR])	283.8 [277.8, 338.1]	339.4 [334.8, 348.6]	283.1 [278.2, 337.0]	296.2 [273.5, 354.6]

## Results

We automatically analyzed the recordings of a total of 13,326 participants. There were 3356 recordings of optimal quality with a minimal length of 300 s. The median recording length in this subset was 339 s (range 300–657 s). Using the complete recording, we found a geometric mean xBRS of 11.6 ms/mmHg (geometric SD 1.8), a mean SDNN of 52.4 ms (SD 23.6), and a mean RMSDD 46.9 ms (SD 28.2). Analysis of the shortened recordings showed that for xBRS and SDNN, there was a poor agreement between records with a length between 30 and 60 s and the complete recording, with ICCs ranging between 0.69 and 0.83. For RMSDD, adequate agreement was already reached after 60 s, with an ICC of 0.94 and difference between − 17.8 and 23.2 ms in the Bland–Altman plot. For xBRS and SDNN, a minimum duration of respectively 120 s and 180 s was required, to reach an ICC > 0.90 (ICC 0.93 for both xBRS and SDNN, supplemental Fig. 2). RMSDD was lower when starting at the end of the recording compared with starting at the beginning with a mean RMSDD of 47.9 ms compared to 45.3 ms (*p* < 0.001) for a 180-s recording (Table 2). A similar, but less pronounced difference was observed for SDNN (51.6 ms vs 49.5 ms; *p* < 0.001) that coincided with an elevated heart rate (63.5 vs 63.8 beats per minute; *p* < 0.001). For xBRS, no systematic difference was observed (*p* = 0.675). In the subset of 100 recordings that were analyzed by hand, we found overall good agreement between the manually selected segments and the complete recording for xBRS (ICC 0.99), SDNN (ICC 0.90), and RMSDD (ICC 1.00); see supplemental Table 1.

**Table 2:** Comparison of xBRS, SDNN, and RMSDD of different recordings lengths. Upper panel shows recordings starting from the beginning of the complete measurement, lower panel recordings from the back of the recoding. Spread denotes range for 95% of the differences. Values for xBRS were log-transformed, and geometric mean, SD, and the ratio are shown. *ICC* intra-class correlation coefficient, *HR* heart rate in beats per minutes, *xBRS* cross-correlation baroreflex sensitivity in ms/mmHg, *SDNN* standard deviation of normal to normal intervals in ms, *RMSDD* the squared root of the mean squared successive difference between adjacent normal-to-normal intervals in ms

Duration	HR	SD	xBRS	SD	Ratio	Spread		ICC	SDNN	SD	Diff	Spread		ICC	RMSDD	SD	Diff	Spread		ICC
Complete	63.7	9.2	11.6	1.8				ref	52.4	23.6				ref	46.9	28.1				ref
	Beginning from start of complete recording																			
30 s	63.0	9.3	10.5	2.2	0.91	0.31	2.66	0.69	47.8	26.8	− 4.5	− 37.8	28.8	0.77	50.2	31.6	3.3	− 23.8	30.5	0.89
60 s	63.2	9.4	10.9	2.0	0.94	0.45	1.98	0.83	49.7	25.5	− 2.7	− 30.5	25.1	0.83	49.6	30.3	2.7	− 17.8	23.2	0.94
120 s	63.4	9.3	11.5	1.9	0.99	0.63	1.55	0.93	50.8	24.6	− 1.6	− 23.1	19.9	0.896	48.6	29.4	1.7	− 12.1	15.6	0.97
180 s	63.5	9.3	11.6	1.9	1.00	0.75	1.34	0.97	51.6	25.7	− 0.7	− 18.2	16.7	0.93	47.9	28.9	1.1	− 8.6	10.7	0.99
240 s	63.6	9.2	11.7	1.8	1.00	0.84	1.20	0.99	51.9	24.7	− 0.4	− 11.6	10.8	0.97	47.5	28.7	0.6	− 6.0	7.2	0.99
300 s	63.6	9.2	11.6	1.8	1.00	0.91	1.11	1.00	52.2	24.1	− 0.1	− 6.7	6.4	0.99	47.1	28.4	0.3	− 3.0	3.6	1.00
	Beginning from end of complete recording																			
30 s	63.9	9.3	11.5	2.0	0.99	0.49	2.02	0.84	43.3	24.2	− 9.1	− 40.6	22.5	0.77	44.2	28.8	− 2.7	− 25.8	20.5	0.91
60 s	63.9	9.3	11.5	1.9	0.99	0.63	1.56	0.93	46.4	23.5	− 6.0	− 32.1	20.2	0.84	44.6	27.9	− 2.2	− 20.9	16.5	0.94
120 s	63.9	9.2	11.5	1.9	0.99	0.72	1.37	0.96	48.5	23.2	− 3.8	− 25.3	17.6	0.89	45.1	28.1	− 1.8	− 15.1	11.4	0.97
180 s	63.8	9.2	11.6	1.9	1.00	0.78	1.29	0.98	49.5	23.0	− 2.8	− 20.6	14.9	0.92	45.3	28.0	− 1.5	− 11.6	8.5	0.98
240 s	63.8	9.2	11.7	1.8	1.01	0.82	1.23	0.99	50.5	23.5	− 1.8	− 14.4	10.7	0.96	45.7	28.1	− 1.2	− 8.6	6.2	0.99
300 s	63.7	9.2	11.7	1.8	1.01	0.88	1.16	0.99	51.4	23.5	− 0.9	− 8.6	6.8	0.99	46.2	28.1	− 0.6	− 4.8	3.6	1.00

### Regression analysis

**Fig. 1:**
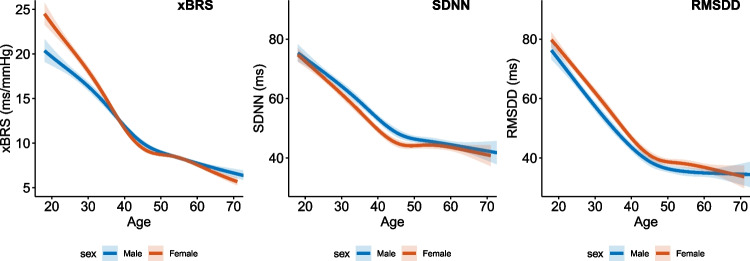
Relation between sex, age, and parameters for sympathovagal balance. Band depicts 95% confidence interval. xBRS, cross-correlation baroreflex sensitivity; SDNN, standard deviation of normal-to-normal intervals; RMSDD, the squared root of the mean squared successive difference between adjacent normal-to-normal intervals. xBRS, SDNN, and RMSDD are all significantly associated with age (*p* < 0.001), with significant interaction between age and sex for xBRS (*p* < 0.001). For SDNN and RMSDD, no interaction with sex was found (respectively *p* = 0.25 and *p* = 0.24)

We included 10,252 (77%) with a minimal recording duration of 180 s, for the regression analysis. A total of 2577 recordings were excluded because the physical calibration criterion was not met, 82 because the recording was shorter than 180 s, and 336 recordings were excluded because ECG data were lacking or did not show sinus rhythm, while 79 recordings were excluded because more than 20% of the beats were removed as a result of excessive variations in the local median IBI (supplementary Fig. 3). In this subset, the median age was 46 years (range 18–73), 54% were women, 33.8% had a history of hypertension, and 14.8% had a CVD history. There were no important differences between the complete cohort and the subset of optimal quality. The distribution of the ethnicities and the use of blood pressure-lowering medication was similar between this final set used for the regression analysis, the subset of optimal quality, and the complete cohort (Table 1). Between ages 18 and 50 years, there was a negative linear association between xBRS, SDNN, and RMSDD and age in both men and women (*p* < 0.001 for all parameters). For xBRS, there was a significant interaction (*p* < 0.001) between sex and age, with a decrease of 4.3 (95%CI 4.0–4.6) ms/mmHg for men and 5.9 (95%CI 5.6–6.1) ms/mmHg for women. For SDNN and RMSDD, we did not find an interaction with sex (*p* = 0.25 and *p* = 0.24), with a decrease of 10.2 (95%CI 9.6–10.8) ms and 13.4 (95%CI 12.7–14.1) ms respectively for every 10 years in both sexes. Above the age of 50, values stabilized to a mean xBRS of 8.8 ms/mmHg, mean SDNN of 45.1 ms, and RMSDD of 36.7 ms for men and women (Fig. 1).

**Fig. 2:**
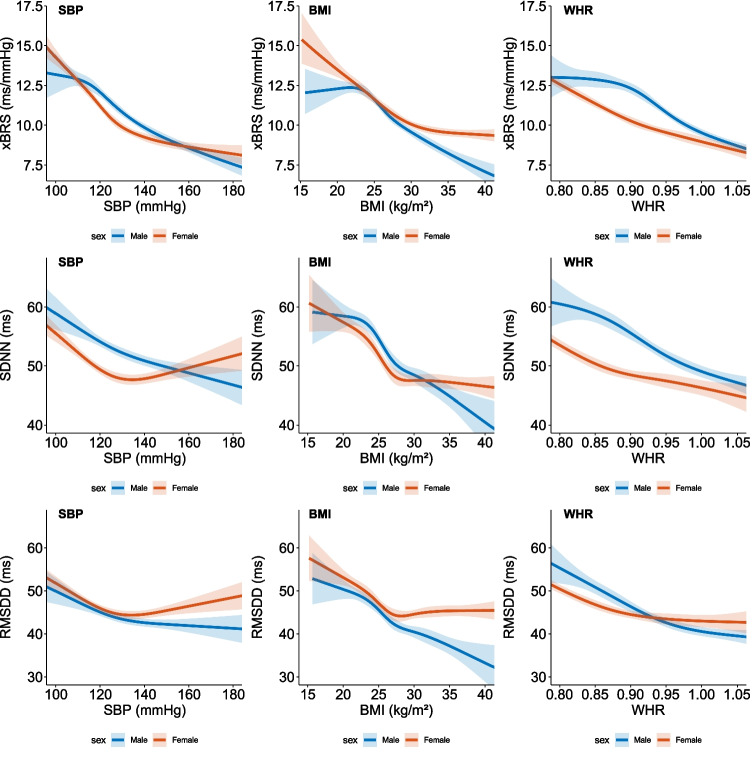
Association between systolic blood pressure (SBP), body mass index (BMI), and WHR (waist-hip ratio) and autonomic function parameters. Line shows results from a regression model, with correction for age, depicted as the predicted value for an age of 45 years. SBP, BMI, and WHR were all significantly associated with xBRS, SDNN, and RMSDD (*p* < 0.001). All depicted models showed a significant interaction with sex (all *p* < 0.001, except *p* = 0.001 for BMI and SDNN, and *p* = 0.03 for SBP and RMSDD). Band depicts 95% confidence interval. xBRS, cross-correlation baroreflex sensitivity; SDNN, standard deviation of normal-to-normal intervals; RMSDD, the squared root of the mean squared successive difference between adjacent normal-to-normal intervals. SBP, BMI, and WHR were all significantly associated with SDNN and RMSDD (*p* < 0.001)

In the regression analyses, we found that increases in SBP, BMI, and WHR were all associated with a decreased xBRS, SDNN, and RMSDD (*p* < 0.001 for all comparisons with correction for age and sex). These relations were all significantly dependent on sex; Fig. 2 shows the correlations using sex-specific splines. For SBP, xBRS decreased linearly in men with a mean of 12.1 (95%CI 11.8–12.4) ms/mmHg at a SBP of 120 mmHg to a mean of 8.56 ms/mmHg (95%CI 8.30–8.83) for a SBP of 160 mmHg. In women however, there was a steep decrease in xBRS with a mean of 14.3 (95%CI 13.8–14.8) ms/mmHg at a SBP of 100 mmHg and a mean xBRS of 9.3 (95%CI 9.0–9.5) ms/mmHg at 140 mmHg; hereafter, xBRS did not decrease further. In women, we found that xBRS decreased up to a BMI of 30 kg/m^2^ with a mean xBRS of 11.6 (95%CI 11.4–11.9) ms/mmHg for a BMI of 25 kg/m^2^ and 10.1 (95%CI 9.9–10.3) ms/mmHg for a BMI of 30 kg/m^2^, followed by a stabilization at increasing BMI. In men, we found that xBRS remained negatively associated with BMI, also in those with BMI > 30 kg/m^2^. In line with this observation, we found that the slope between WHR and xBRS tended to stabilize in women in WHR for values above 0.90. For SDNN and RMSDD, we found similar relations with SBP, BMI, and WHR.

## Discussion

We constructed and applied an algorithm for automated analysis of autonomic function, calculating xBRS and HRV from continuous finger blood pressure recordings. This enables the determination of key parameters for autonomic function in large-scale studies. We show that a time period of at least 180 s is necessary to reliably assess xBRS and HRV, with an ICC > 0.90 compared to the full recording. Forward and backward analyses of shortened recordings showed no significant differences in xBRS, but revealed a minor increase in heart rate and decrease in SDNN and RMSDD after 10 min of supine rest. Given the small difference at the end of both time courses, we estimate that this time period is sufficient for equilibration. The main reason for not meeting the quality criteria was failure to reach a stable segment without automatic internal calibration. Through our large-scale automated analysis, we show that xBRS, SDNN, and RMSDD are similar in men and women and decrease linearly with age and that above 50 years of age the decline is less steep. Additionally, we show that in women xBRS and HRV tend to stabilize at higher BP, BMI, and WHR, while xBRS and HRV progressively decrease in men.

We show that the algorithm gives consistent results when using different segments of the recording. We observed that a recording time of 60 s was sufficient for RMSDD as it reflects short-term variations in HRV. For SDNN, which includes longer variations in HRV, we observed a systemic bias of lower values in shorter recordings, and we found that a minimum length of 180 s was required to optimize accuracy. This is in line with earlier findings by Munoz et al. and challenges the recommendation in current guidelines that a minimum recording length of 300 s is required for reliable assessment of HRV [11, 17]. We found no differences in average xBRS for recordings of different lengths, but observed that a duration of 120 s was required to optimize precision as xBRS consists of averaged 10-s intervals. HRV was 5% lower in recordings starting from the end. By this time, heart rate values were slightly higher, which may be provoked by increasing restlessness at the end of the period of supine rest. It is well known that changes in sympathovagal balance as induced by changes in body position or exercise level have a direct effect on the outcome parameters [18]. In the manually analyzed recordings, we obtained slightly lower values for SDNN and RMSDD, which can be attributed to a reduction in overall variation when manually selecting a stable segment of the complete recording further emphasizing the importance of obtaining recordings under standardized conditions. We found no relevant differences in baseline characteristics between the complete cohort, the subset with optimal quality, and the subset with recordings of sufficient quality, supporting the applicability of our technique. We observed some differences in the distribution of the different ethnic groups, but these can be attributed to logistical reasons, as not all ethnic groups were included at the same time in the study.

In our large-scale analysis of xBRS and HRV, we show that all autonomic parameters decreased linearly with age up to age 50 years, where after xBRS and HRV stabilized in both men and women. For xBRS, we found that the decrease with age was steeper in women, while we did not find important differences in the slope for SDNN and RMSDD. Earlier analyses of the RMSDD from 10-s ECG recordings from the Lifelines cohort and for the SDNN using 5-min ECG recording from the KORA S4 cohort revealed a similar pattern [19, 20]. In the KORA S4-cohort, a linear relationship for SDNN with age was reported in healthy individuals without hypertension and cardiovascular disease aged between 18 and 60 years; however, in contrast to that study, we did not find an interaction with sex in our analysis. The decrease in HRV has been attributed to age-dependent alterations in heart rate control as a result of decreasing vagal activity with cardiovascular aging [21, 22]. For BRS, earlier studies have shown a negative correlation with age. Ebert has shown a linear decrease of cardiac BRS measured using pharmacologically induced BP changes in 66 individuals aged between 18 and 71 years [23]. More recently, using frequency domain analysis of spontaneous variations in BP and heart rate, a similar relation with age was observed in 110 apparently healthy individuals aged between 21 and 70 years [24]. However, in our large-scale population-based analysis, we observed a non-linear relation with an inflection point around 50 years, which may relate to similar mechanisms involving heart rate variability as xBRS correlates changes in heart rate to changes in blood pressure. Indeed, it is well-established that respiratory sinus arrhythmia, which—like xBRS—is predominantly mediated through vagal control, diminishes with age [25, 26].

In the regression analysis, we observed that xBRS and HRV progressively decreased for an increase in SBP, BMI, and WHR in men. However, in women, both xBRS and HRV did not further decrease at higher BP, BMI, and WHR. Earlier studies using autonomic tests with vasoactive drugs have shown that the contribution of the sympathetic nervous system in blood pressure control is larger in men compared to women, which could explain the present findings [27]. Sex differences in the association with BMI can, in part, be explained by differences in body composition between men and women [28]. Taking the sex-specific normal values for WHR of 0.90 for men and 0.85 for women into account, we found similar differences for the relation with WHR, suggesting that this relation is intrinsically different between men and women [29].

Earlier studies have shown that both HRV and BRS predict future cardiovascular events and cardiac mortality in patients with heart failure and coronary artery disease [30, 31]. A recent meta-analysis further showed that HRV is an important predictor for the first cardiovascular event in both women and men [1]. The observed, but disparate, associations of SBP and body composition with sympathovagal balance in men and women in our population support these results. Whether the observed disparities in the association between sympathovagal balance and CVD risk factors between men and women also translate in differences in the predictive value of cardiovascular morbidity and mortality remains to be determined. 

A strength of this study is the use of a large multi-ethnic population-based cohort study for both the development of the algorithm and the cross-sectional analysis. This allowed us to perform an initial analysis on a sufficiently large dataset of optimal quality. The results from the regression analysis in the large cohort of 11,000 participants support the applicability of our algorithm. Also, data on combined xBRS and HRV assessment across the complete age range, in particular in younger (< 30 years) and older (> 60 years) adults, is to our knowledge still lacking. Limitations of our study are its cross-sectional design and the lack of ECG-based recordings, which are considered to be the gold standard of HRV and BRS assessment. Earlier studies have shown that the inter-beat interval derived from finger blood pressure recordings has excellent correspondence compared to the timing derived from the electrocardiogram at rest [32–34]. In addition, we used an automatic filter to exclude abnormal or ectopic beats. However, the lack of ECG data prevented us from manually excluding ectopic beats based on their electrocardiographic morphology. We did however exclude recordings of individuals who were not in sinus rhythm on the ECG which had been performed at the same visit. In addition, we manually verified the automatic filtering of the recording against manual analysis of HRV and xBRS in 100 recordings, which showed overall good agreement. Finally, the gold standard for assessment of BRS consists of measurement after induction of blood pressure changes using vasoconstricting drugs, which is not feasible to perform in large-scale studies. A recent study has validated the xBRS against these measurements [6].

In conclusion, we have constructed an algorithm to automatically determine sympathovagal balance based on continuous finger blood pressure measurements that can be used in large-scale population cohort studies. Our large-scale analysis of xBRS and HRV reveals important relations between changes in autonomic function parameters and changes in age and other relevant anthropometric parameters. Furthermore, our findings show important sex differences in relation with blood pressure and body mass composition, pointing towards possible differences in the pathogenesis of cardiovascular complications. Our newly developed methodology can aid in automatically and systematically performing these measurements at large scale. Future large-scale studies could focus on unravelling the nature of differences in sympathovagal balance and its association with CVD risk and its predictive value for CVD outcomes in different populations.

## Supplementary Information

Below is the link to the electronic supplementary material.

Supplementary file1 (DOCX 796 kb)

Supplementary file2 (ZIP 48.4 kb)

## References

[1] Hillebrand S, Gast KB, De Mutsert R (2013). Heart rate variability and first cardiovascular event in populations without known cardiovascular disease: meta-analysis and dose-response meta-regression. Europace.

[2] Singh JP, Larson MG, Tsuji H (1998). Reduced heart rate variability and new-onset hypertension: insights into pathogenesis of hypertension: The Framingham Heart Study. Hypertension.

[3] Schroeder EB, Liao D, Chambless LE (2003). Hypertension, blood pressure, and heart rate variability: the Atherosclerosis Risk in Communities (ARIC) study. Hypertension.

[4] Grassi G, Seravalle G, Dell’Oro R (2000). Adrenergic and reflex abnormalities in obesity-related hypertension. Hypertension.

[5] Sajadieh A, Nielsen OW, Rasmussen V (2004). Increased heart rate and reduced heart-rate variability are associated with subclinical inflammation in middle-aged and elderly subjects with no apparent heart disease. Eur Heart J.

[6] Wesseling KH, Karemaker JM, Castiglioni P (2017). Validity and variability of xBRS: instantaneous cardiac baroreflex sensitivity. Physiol Rep.

[7] Snijder MB, Galenkamp H, Prins M (2017). Cohort profile: The Healthy Life in an Urban Setting (HELIUS) study in Amsterdam, the Netherlands. BMJ Open.

[8] The Task Force for the management of arterial hypertension of the European Society of Cardiology (ESC) and the European Society of Hypertension (ESH) (2018). 2018 ESC/ESH Guidelines for the management of arterial hypertension. Eur Heart J.

[9] ter Haar CC, Kors JA, Peters RJG, et al (2020) Prevalence of ECGs exceeding thresholds for ST-segment–elevation myocardial infarction in apparently healthy individuals: the role of ethnicity. J Am Heart Assoc 9:e015477. 10.1161/JAHA.119.015477 10.1161/JAHA.119.015477 PMC7670498 32573319

[10] BMEYE Nexfin HD: Operator’s manual

[11] (1996) Heart rate variability. Standards of measurement, physiological interpretation, and clinical use. Eur Heart J 17:354–381. 10.1161/01.CIR.93.5.1043 8737210

[12] Westerhof BE, Gisolf J, Stok WJ (2004). Time-domain cross-correlation baroreflex sensitivity: performance on the EUROBAVAR data set. J Hypertens.

[13] Martin Bland J, Altman D (1986). Statistical methods for assessing agreement between two methods of clinical measurement. Lancet.

[14] Cicchetti DV (1994). Guidelines, criteria, and rules of thumb for evaluating normed and standardized assessment instruments in psychology. Psychol Assess.

[15] Koo TK, Li MY (2016). A guideline of selecting and reporting intraclass correlation coefficients for reliability research. J Chiropr Med.

[16] Sloan RP, Huang MH, McCreath H (2008). Cardiac autonomic control and the effects of age, race, and sex: the CARDIA study. Auton Neurosci Basic Clin.

[17] Munoz ML, Van Roon A, Riese H (2015). Validity of (ultra-)short recordings for heart rate variability measurements. PLoS One.

[18] Westerhof BE, Gisolf J, Karemaker JM (2006). Time course analysis of baroreflex sensitivity during postural stress. AJP Hear Circ Physiol.

[19] Tegegne BS, Man T, van Roon AM (2018). Determinants of heart rate variability in the general population: the Lifelines Cohort study. Hear Rhythm.

[20] Voss A, Schroeder R, Heitmann A (2015). Short-term heart rate variability - influence of gender and age in healthy subjects. PLoS One.

[21] Agelink MW, Malessa R, Baumann B (2001). Standardized tests of heart rate variability: normal ranges obtained from 309 healthy humans, and effects of age, gender, and heart rate. Clin Auton Res.

[22] Ferrari AU (2002) Modifications of the cardiovascular system with aging. Am J Geriatr Cardiol 11:30–33+39. 10.1111/1467-8446.00044-i1 10.1111/1467-8446.00044-i1 11773713

[23] Ebert TJ, Morgan BJ, Barney JA et al (1992) Effects of aging on baroreflex regulation of sympathetic activity in humans. Am J Physiol - Hear Circ Physiol 263:. 10.1152/ajpheart.1992.263.3.h798 10.1152/ajpheart.1992.263.3.H798 1415605

[24] Milan-Mattos JC, Porta A, Perseguini NM (2018). Influence of age and gender on the phase and strength of the relation between heart period and systolic blood pressure spontaneous fluctuations. J Appl Physiol.

[25] Hrushesky WJM, Fader D, Schmitt O, Gilbertsen V (1984) The respiratory sinus arrhythmia: a measure of cardiac age. Science (80- ) 224:1001–1004. 10.1126/science.6372092 10.1126/science.6372092 6372092

[26] Yasuma F, Hayano JI (2004). Respiratory sinus arrhythmia: why does the heartbeat synchronize with respiratory rhythm?. Chest.

[27] Christou DD, Jones PP, Jordan J (2005). Women have lower tonic autonomic support of arterial blood pressure and less effective baroreflex buffering than men. Circulation.

[28] Stefan N (2020). Causes, consequences, and treatment of metabolically unhealthy fat distribution. Lancet Diabetes Endocrinol.

[29] World Health Organisation (WHO) (2008) WHO | Waist Circumference and Waist–Hip Ratio. Report of a WHO Expert Consultation. Geneva, 8–11 December 2008. 8–11

[30] La Rovere MT, Bigger JT, Marcus FI (1998). Baroreflex sensitivity and heart-rate variability in prediction of total cardiac mortality after myocardial infarction. Lancet.

[31] Pinna GD, Maestri R, Capomolla S (2005). Applicability and clinical relevance of the transfer function method in the assessment of baroreflex sensitivity in heart failure patients. J Am Coll Cardiol.

[32] McKinley PS, Shapiro PA, Bagiella E (2003). Deriving heart period variability from blood pressure waveforms. J Appl Physiol.

[33] Schäfer A, Vagedes J (2013). How accurate is pulse rate variability as an estimate of heart rate variability?: A review on studies comparing photoplethysmographic technology with an electrocardiogram. Int J Cardiol.

[34] Pernice R, Javorka M, Krohova J (2019). Comparison of short-term heart rate variability indexes evaluated through electrocardiographic and continuous blood pressure monitoring. Med Biol Eng Comput.

