# Supplementary material for: Automated analysis of finger blood pressure recordings provides insight in determinants of baroreflex sensitivity and heart rate variability—the HELIUS study
Source: Med Biol Eng Comput. 2023 Jan 23;61(5):1183–91. doi: 10.1007/s11517-023-02768-4 (PMC10083154; doi:10.1007/s11517-023-02768-4)
Supplement: Supplementary file 1 — (DOCX 796 kb) [file 11517_2023_2768_MOESM1_ESM.docx]

**SUPPLEMENTAL MATERIAL**

**Automated analysis of finger blood pressure recordings provides insight in determinants of baroreflex sensitivity and heart rate variability - the HELIUS study**D. Collard^a^, B.E. Westerhof^b,c^, J.M. Karemaker^c^, W.J. Stok^c^, P.G. Postema^d^, C.T.P. Krediet^e^, L. Vogt^e^, B.J.H. van den Born^a,f^

### Author affiliations:

^a^ Amsterdam UMC, University of Amsterdam, Department of Internal Medicine, Section Vascular Medicine, Amsterdam Cardiovascular Sciences, Amsterdam, The Netherlands

^b^ Amsterdam UMC, Vrije Universiteit Amsterdam, Department of Pulmonary Medicine, Amsterdam, The Netherlands

^c^ Amsterdam UMC, University of Amsterdam, Department of Medical Biology, section Systems Physiology, Amsterdam, The Netherlands

^d^ Amsterdam UMC, University of Amsterdam, Heart Center, Department of Cardiology, Amsterdam Cardiovascular Sciences Amsterdam, The Netherlands

^e^ Amsterdam UMC, University of Amsterdam, Department of Internal Medicine, Section Nephrology, Amsterdam, Amsterdam Cardiovascular Sciences, The Netherlands

^f^ Amsterdam UMC, University of Amsterdam, Department of Public and Occupational Health, Amsterdam Public Health Research institute, Amsterdam, The Netherlands

**Supplemental figure 1:** Schematic overview of the algorithm used for derivation of cross correlation baroreflex sensitivity (xBRS) and heart rate variability (HRV).

**
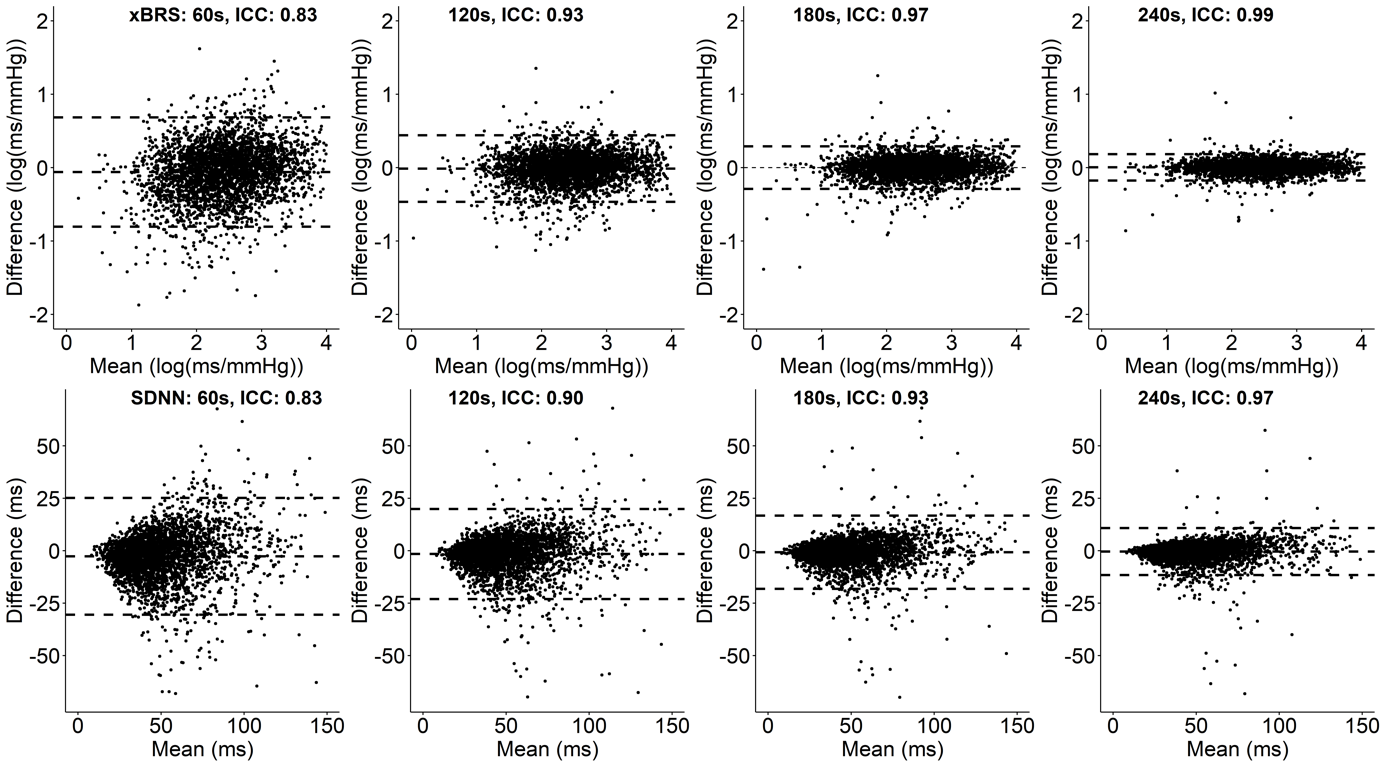
**

**Supplemental figure 2**: Bland-Altman plots for different recording lengths. Dotted lines indicate mean differences and spread of 95% of the differences. ICC the intra class correlation coefficient. xBRS: cross-correlation baroreflex sensitivity, SDNN standard deviation of normal to normal intervals. Values for xBRS were log-transformed and depicted as transformed values.

**
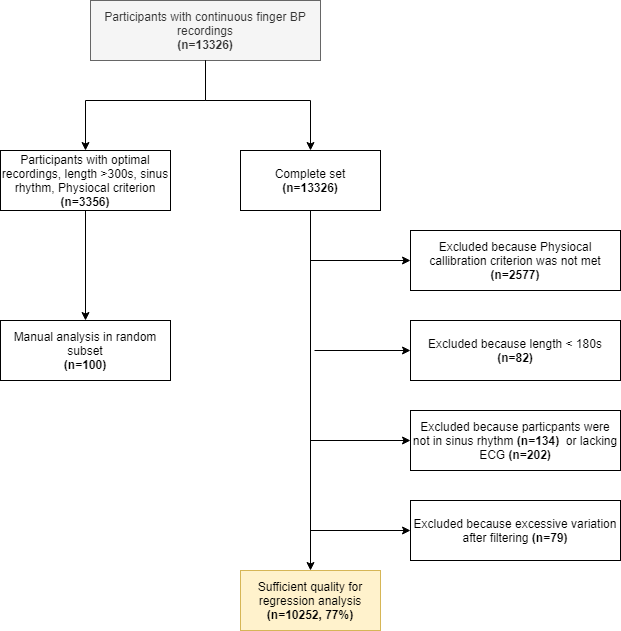
**

**Supplemental figure 3**: Flowchart depicting selection of participants with a recording of optimal quality for analysis of minimum required duration and selection of participants with recording of sufficient quality for regression analysis.

| **Duration** | **HR** | **SD** | **xBRS** | **SD** | **Ratio** | **Spread** | | **ICC** | **SDNN** | **SD** | **Diff** | **Spread** |  | **ICC** | **RMSDD** | **SD** | **Diff** | **Spread** |  | **ICC** |
| --- | --- | --- | --- | --- | --- | --- | --- | --- | --- | --- | --- | --- | --- | --- | --- | --- | --- | --- | --- | --- |
| Automatic | 62.6 | 9.9 | 11.2 | 2.0 |  |  |  | ref | 57.8 | 32.3 |  |  |  | ref | 53.4 | 52.9 |  |  |  | ref |
| Manual | 62.7 | 9.9 | 11.5 | 2.0 | 1.03 | 0.90 | 1.18 | 0.99 | 53.5 | 31.0 | -4.2 | -31.4 | 22.9 | 0.904 | 52.0 | 53.8 | -1.4 | -10.2 | 7.4 | 1.00 |

**Supplemental table 1:** Comparison of xBRS, SDNN, RMSDD between the automatically and manually analyzed recording. Mean recording length was 269s in manually analyzed segments; 345s in automatically analyzed recordings. Spread denotes range for 95% of the differences. Values for xBRS were log-transformed; and geometric mean, SD, and the ratio are shown. ICC: intra-class correlation coefficient, HR: heart rate in beats per minutes, xBRS: cross-correlation baroreflex sensitivity in ms/mmHg, SDNN standard deviation of normal to normal intervals in ms, RMSDD: the squared root of the mean squared successive difference between adjacent normal-to-normal intervals in ms.
